# An RND-Type Efflux System in *Borrelia burgdorferi* Is Involved in Virulence and Resistance to Antimicrobial Compounds

**DOI:** 10.1371/journal.ppat.1000009

**Published:** 2008-02-29

**Authors:** Ignas Bunikis, Katrin Denker, Yngve Östberg, Christian Andersen, Roland Benz, Sven Bergström

**Affiliations:** 1 Department of Molecular Biology, Umeå University, Umeå, Sweden; 2 Laboratory for Molecular Infection Medicine Sweden (MIMS), Umeå University, Umeå, Sweden; 3 Lehrstuhl für Biotechnologie, Biozentrum der Universität Würzburg, Würzburg, Germany; Children's Hospital Boston, United States of America

## Abstract

*Borrelia burgdorferi* is remarkable for its ability to thrive in widely different environments due to its ability to infect various organisms. In comparison to enteric Gram-negative bacteria, these spirochetes have only a few transmembrane proteins some of which are thought to play a role in solute and nutrient uptake and excretion of toxic substances. Here, we have identified an outer membrane protein, BesC, which is part of a putative export system comprising the components BesA, BesB and BesC. We show that BesC, a TolC homolog, forms channels in planar lipid bilayers and is involved in antibiotic resistance. A besC knockout was unable to establish infection in mice, signifying the importance of this outer membrane channel in the mammalian host. The biophysical properties of BesC could be explained by a model based on the channel-tunnel structure. We have also generated a structural model of the efflux apparatus showing the putative spatial orientation of BesC with respect to the AcrAB homologs BesAB. We believe that our findings will be helpful in unraveling the pathogenic mechanisms of borreliae as well as in developing novel therapeutic agents aiming to block the function of this secretion apparatus.

## Author Summary

Lyme disease is caused by infection with the spirochete *Borrelia burgdorferi*. These spirochetes cycle between *Ixodes* ticks and vertebrate reservoirs, mainly rodents, but also birds. Previous studies have revealed major differences in the *B. burgdorferi* cell envelope structure and membrane composition compared to those of other bacteria. Proteins embedded in the bacterial membranes fulfill a number of tasks that are crucial for bacterial cells, such as solute and protein transport, as well as signal transduction, and interaction with other cells. Microorganisms have evolved mechanisms to protect themselves against harmful substances and secrete these through efflux pumps. So far, little is known about mechanisms of drug efflux systems in borreliae. Herein we identified an outer membrane channel forming protein important for *B. burgdorferi* to cause infection in mice and that also is involved in antibiotic resistance. We believe that this work will be helpful to understand the mechanisms underlying borreliae infection biology as well as in developing new therapeutic agents aiming to block this multi drug efflux system.

## Introduction

*Borrelia burgdorferi* was described as a causative agent of Lyme borreliosis in the early eighties [1],[2]. The bacterium can be transmitted to humans by the bite of an infected tick of the genus *Ixodes* and cause Lyme borreliosis resulting in a wide variety of clinical manifestations [3]. The bacterium can survive for an extended time in humans by evading the immune system, and leading to chronic infection with arthritis, dermatitis or neuroborreliosis. To date, antibiotic treatment with tetracycline or β-lactam is successful in most cases [3], especially when infection is diagnosed at an early stage. However, *B. burgdorferi* has natural resistance towards several antibiotics such as phosphomycin and sulfamethoxazole. The mechanism(s) of resistance are unknown but may include those already described for other bacteria, such as enzymes, mutation of the antibiotic target, or efflux pumps.

In comparison to enteric Gram-negative bacteria, the density of transmembrane proteins in *B. burgdorferi* is low [4]. Nevertheless, among spirochetes *B. burgdorferi* has the highest concentration of transmembrane proteins [5],[6]. Porins are one class of transmembrane proteins responsible for solute transport across the outer membrane of Gram-negative bacteria. Integral outer membrane proteins with functions similar to porins have been reported for *Borrelia* [7],[8],[9],[10].

During the course of evolution, bacteria have been exposed to a variety of toxic compounds such as toxins, endogenous metabolic end products, and antibiotics. To protect themselves, microorganisms have evolved devices to detoxify and secrete these substances. Bacterial resistance to many classes of antibiotics is provided mainly by membrane transporter proteins called drug efflux pumps [11], which are part of the multi-drug resistance (MDR) efflux systems. Five main families of bacterial MDR transporters have been identified: MF (major facilitator), MATE (multi-drug and toxic efflux), ABC (ATP binding cassette), SMR (small multi-drug resistance), and RND (resistance-nodulation-division) [12]. RND transporters exist in all kingdoms of living organisms, but seem to be involved in drug resistance especially in Gram-negative bacteria where they export toxic substances across both membranes of the cell envelope in a single energy-coupled step [13]. These efflux pumps are made of three components: a cytoplasmic membrane export system that acts as an energy-dependent pump, a membrane fusion protein (MFP), and an outer membrane factor (OMF) [13],[14]. The major antibiotic efflux activity of this type in *Escherichia coli* is mediated by the tripartite multi-drug resistance pump AcrAB-TolC [15],[16] which transports substrates from the cell into the external medium, bypassing the periplasm and the outer membrane [17]. This complex consists of the inner membrane translocase AcrB which is thought to be a proton transporter [18],[19] belonging to the resistance-nodulation-division (RND) family of proteins [20], the outer membrane channel TolC, which is an OMF [21], and a periplasmic linker protein, AcrA, which is a member of the membrane fusion protein (MFP) superfamily [22]. TolC forms trimers, in which each monomer contributes four β-strands to a single channel-forming unit. TolC homologues are ubiquitous among Gram-negative bacteria, and thus far nearly a hundred have been identified [14].

In this study, we identified a channel-forming activity corresponding to a TolC homolog, BesC, in the *B. burgdorferi* outer membrane. We show that the BesC protein is necessary for *B. burgdorferi* to establish infection in mice and is involved in antibiotic resistance. Furthermore, we determined the biophysical properties of the channel formed by BesC and generated a model of the putative efflux apparatus.

## Results

### *In silico* identification of a *Borrelia* TolC homolog

In a comprehensive database search for outer membrane porins of different Gram-negative bacteria Yen *et al.* predicted that the *Borrelia burgdorferi* B31 genome harbors a putative member of an outer membrane factor (OMF) family hypothetical protein designated BB0142 [23]. We analyzed the amino acid sequence of this protein using an NCBI conserved domain search which revealed significant similarity (E value  =  5^−22^) to proteins of the outer membrane efflux protein (OEP) family, including the *E. coli* outer membrane protein TolC [24]. This is an important indication that BB0142 is a TolC homologue. The *Borrelia* genome contains only one gene encoding a protein belonging to the OEP family. Investigation of the genes directly flanking *bb0142* showed that *bb0140* codes for AcrB-like protein, an inner membrane transporter of the RND (resistance, nodulation, cell-division) family, and *bb0141* codes for an AcrA-like protein, an adaptor protein also known as membrane fusion protein. Homologs of these proteins form tripartite multi-drug efflux pumps in many Gram-negative bacteria. Therefore, we propose renaming these genes *besA (bb0141)*, *besB (bb0140)* and *besC (bb0142)* for ***B*** *orrelia* **e**fflux **s**ystem proteins A, B and C.

### *besB*, *besA*, and *besC* are co-transcribed

**Figure 1:**
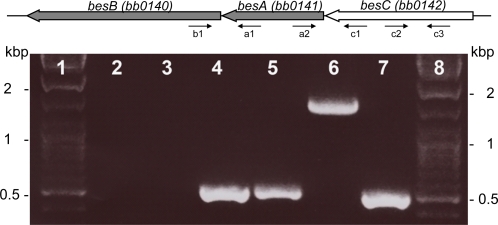
Schematic representation of the putative *besB-besA-besC* operon in *Borrelia burgdorferi* genome and results from the reverse transcription PCR. Lane 1 and 8; molecular weight markers, lane 2; PCR reaction without template using primer pair c2 and c3 served as negative control, lane 3; primer pair c2 and c3 in ordinary PCR reaction using RNA as template served as negative control for DNA contamination, lane 4; primer pair b1 and a1, lane 5; primer pair a2 and c1, lane 6; primer pair b1 and c1, lane 7; primer pair c2 and c3 served as positive control.

According to the *B. burgdorferi* genome sequence, the genes *besB (bb0140)*, *besA (bb0141)* and *besC (bb0142)* are oriented in the same direction, and separated by 18 and 8 bp, respectively. In order to determine whether the three genes are transcribed as a single transcript, we analyzed RNA from low-passage strain B31 by RT-PCR using primers specific for different genes (Table S1). The primer pair spanning junction of *besB* and *besA* amplified a fragment (lane 4 in Figure 1) demonstrating that both genes are located on the same transcript. Similar results were obtained for primer pair spanning junction of *besA* and *besC* genes (lane 5 in Figure 1). Therefore we conclude that all three genes are transcribed together. This was further confirmed by amplifying a product spanning *besB*, *besA* and *besC* using primers specific for *besB* and *besC* (lane 6 in Figure 1). Negative control reactions, in which reverse transcriptase was omitted, yielded no such product (lane 3 in Figure 1), indicating that the products were derived from RNA rather than contaminating DNA. Additionally, *besC*-specific primers were used as a positive control (lane 7 in Figure 1). These data show that *besB*, *besA* and *besC* are transcribed as a single transcript.

### Inactivation and complementation of the *besC* gene in *B. burgdorferi*

**Figure 2:**
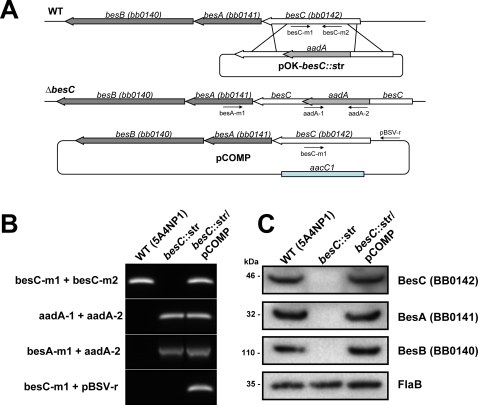
Characterization of the *besC* mutant strains. (A) Schematic representation of *besC*, *becA* and *besB* genes in the *Borrelia burgdorferi* B31 chromosome, insertion of the *aadA* gene cassette by homologous recombination and complementation plasmid. Arrows indicate the relative positions of the oligonucleotides used. The diagram is not drawn to scale. (B) PCR analysis of the wild-type strain, the resulting *besC* mutant and the complemented strain using primer pairs specific either only for the *besC* and *aadA* genes; or one primer specific for *besA* and the other for *aadA* gene. The PCR was also used to confirm the presence of the shuttle vector in the complemented strain using one primer specific for *besC* and another specific for the shuttle vector. (C) Immunoblotting using antiserum raised against BesA, BesB and BesC to determine the presence or absence of these proteins in wild-type, *besC* mutant and the complemented strain.

In order to investigate the potential involvement of BesC in antibiotic resistance and virulence of *B. burgdorferi*, we constructed a *besC* mutant. A streptomycin resistance gene was inserted into the *besC* gene (Figure 2A) of low-passage infectious *B. burgdorferi* strain 5A4NP1 by electroporation with plasmid pOK-*besC::str* as described in Materials and Methods. Plasmid pCOMP (Figure 2A) was used to complement *besC* mutant. Strains were analyzed by PCR to confirm inactivation and complementation of *besC* (Figure 2B).

The expression of BesC protein in strain 5A4NP1, the *besC* mutant and the complemented strain was analyzed by immunoblotting with antiserum raised against the recombinant construct of BesC. As expected, BesC was present in the wild-type and complemented strains, but not in the mutant *besC::str* (Figure 2C). Due to the finding that *besC* is co-transcribed with *besA* and *besB*, membranes were further probed with antibodies raised against recombinant parts of BesA and BesB to determine whether protein expression is also linked. The putative membrane fusion protein BesA and putative inner membrane transporter BesB were expressed in low-passage infectious strain 5A4NP1, as well as in the complemented strains, but neither protein could be detected in the *besC::str* mutant (Figure 2C). This observation further confirmed the RT-PCR results indicating that the three genes are transcriptionally linked.

No phenotypic differences between 5A4NP1 and *besC::str* mutant were observed when the morphology of the spirochetes was compared by microscopy. Growth curves were determined for the 5A4NP1, *besC::str* and the complemented strains under normal growth conditions in liquid medium by counting the spirochetes in a Petroff-Hausser chamber daily. No significant effect on growth could be observed (data not shown).

### Involvement of BesC in antibiotic resistance

It has been shown previously that the outer membrane components are essential for functionality of multi-drug efflux pumps [25]. Therefore susceptibilities of *B. burgdorferi* strain 5A4NP1, *besC::str* mutant strain and the complemented strain to different antimicrobials were tested *in vitro*. In summary, both the MIC (minimal inhibitory concentration) and MBC (minimal borreliacidal concentration) values of the different compound tested for *B. burgdorferi* carrying inactive *besC* were 2- to 64-fold lower as compared to the parental strain 5A4NP1 (Table 1). The MIC and MBC values were essentially the same for the parental strain and complemented mutant (Table 1). Our results therefore suggest that BesC is involved in antibiotic resistance in *B. burgdorferi*.

**Table 1:** *In vitro* antibiotic susceptibility of *B. burgdorferi*.

Strain^a^	Penicillin G^b^		Carbenicillin		Tetracycline		Azithromycin		Cefotaxime		Ethidium bromide		SDS	
	MIC	MBC	MIC	MBC	MIC	MBC	MIC	MBC	MIC	MBC	MIC	MBC	MIC	MBC
WT (5A4NP1)	0.63	20	0.16	1.25	0.31	2.50	0.016	0.125	0.05	0.78	1.56	6.25	781.25	781.25
*besC::str*	0.31	5	0.08	0.31	0.04	0.16	0.002	0.031	0.01	0.20	0.05	0.39	12.21	12.21
*besC::str* + *besC*	0.63	20	0.16	1.25	0.31	2.50	0.016	0.125	0.05	0.78	1.56	6.25	781.25	781.25

a Strains used are described in Materials and Methods.

b The MIC and MBC values for the antimicrobial agents are in µg.

### BesC is essential for mouse infection

To investigate the influence of *besC* inactivation on *B. burgdorferi* virulence, animal infectivity experiments were performed. C3H/HeN mice were infected with strain 5A4NP1, *besC::str* and complemented strain *besC::str* + *besC*. After two weeks heart, bladder, knee and ear were transferred to fresh BSKII media. Cultures were monitored for 4 weeks for spirochete growth. The findings are summarized in Table 2. As expected, cultures from positive control mice contained vigorously growing bacteria. In cultures from mice infected with *besC* mutant no bacterial growth occurred, suggesting that BesC is important for *Borrelia* to establish infection in mice. Furthermore, the complemented strain could infect mice and was re-isolated from all cultured organs. *In vitro* propagation of *B. burgdorferi* strains can result in loss of plasmids; therefore both pre- and post-infection strains were examined for plasmid content in order to confirm that loss of infectivity of *besC::str* strain was not due to loss of important plasmids but due to inactivation of *besC*. All strains contained an intact set of extragenomic elements (data not shown). The presence of the shuttle vector used for complementation in strain *besC::str* + *besC* recovered from infected mice organs was confirmed by PCR as well as by transforming total DNA from this strain into *E. coli* cells and isolating gentamycin-resistant colonies. Subsequent screening of colonies by PCR using primers specific for the shuttle vector confirmed the presence of the pCOMP plasmid (data not shown).

**Table 2:** Mouse infection study using *B. burgdorferi* wild-type, *besC::str* mutant and complemented strains.

Strain	Re-isolation from tissues				No. of mice infected/total
	Heart	Bladder	Knee	Ear	
WT (5A4NP1)	7/7	7/7	7/7	7/7	7/7
*besC::str*	0/7	0/7	0/7	0/7	0/7
*besC::str* + *besC*	7/7	7/7	7/7	7/7	7/7

### Identification of BesC channel-forming activity in the *B. burgdorferi* outer membrane fraction

P66 is a channel-forming protein with a single channel conductance of 11 nS in 1 M KCl, making it the largest outer membrane channel present in *B. burgdorferi* [10]. Absence of the giant P66 outer membrane channel represents a considerable advantage for purification of other *B. burgdorferi* channel-forming proteins [26]. Therefore, strain *p66::str* was used to separate outer membrane proteins by anion exchange chromatography. Proteins were eluted with buffer supplemented with increasing concentrations of NaCl and analyzed in lipid bilayer assays to detect channel-forming activity. The chromatogram taken at 280 nm showed a small protein peak for the fractions eluted at 250 mM NaCl (data not shown), which showed uniform channel-forming activity with a single-channel conductance of about 300 pS. To analyze its protein content, the active fraction was precipitated and separated by SDS-PAGE. Silver stained protein bands were analyzed by mass spectrometry and identified by peptide mass fingerprinting (Figure S1 and Table S2). A protein band with an apparent molecular mass of 48 kDa was identified as BesC, which was verified by Western blot (data not shown). Addition of polyclonal BesC antibodies to the FPLC fraction inhibited channel-forming activity in a lipid bilayer assay, further confirming that BesC is responsible for the channel-forming activity.

To further confirm our findings we analyzed outer membrane fraction of *p66::str/besC::kan* mutant strain separated by anion exchange chromatography as described above. No channel-forming activity with a single-channel conductance of 300 pS could be observed.

### Channel-forming properties of purified BesC

**Figure 3:**
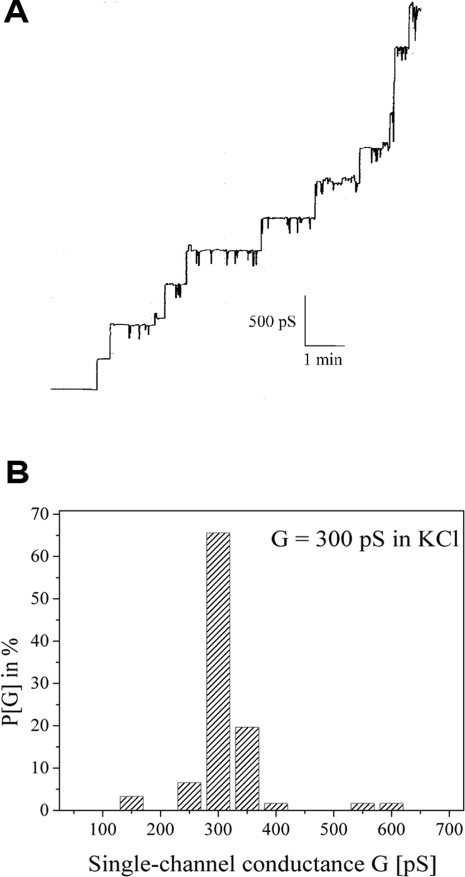
Channel-forming activities of FPLC purified BesC. (A) Single-channel recording of a diphytanoyl phosphatidylcholine/*n*-decane membrane observed in the presence of 100 ng/ml of fraction 35 of the MonoQ-FPLC of the B-fraction of *B. burgdorferi* B31-A *p66::str*. The aqueous phase contained 1 M KCl; V_m_  =  20 mV; T  =  20°C. (B) Histogram of the probability P(G) for the occurrence of a given conductivity unit observed with membranes formed of 1% diphytanoyl phosphatidylcholine/n-decane in the presence of fraction 35 of the MonoQ-FPLC of the B-fraction of *B. burgdorferi* B31-A *p66::str*. P(G) is the probability that a given conductance increment G is observed in the single-channel experiments. It was calculated by dividing the number of fluctuations with a given conductance increment by the total number of conductance fluctuations. The average single-channel conductance for 122 single-channel events was 300 pS.

To analyze the channels formed by BesC, single-channel experiments were performed with purified protein. FPLC fractions containing BesC resulted in a step-wise increase of the membrane conductance in a lipid bilayer assay. Figure 3A shows a single-channel recording of a lipid bilayer membrane in the presence of a very low concentration of BesC at a membrane voltage of 20 mV. Figure 3B shows a histogram of 122 conductance steps observed in single-channel experiments with BesC-containing FPLC fractions. This protein forms obvious channels with an average single-channel conductance of 300 pS in 1 M KCl. Interestingly the 300 pS pore did not display any voltage dependence even at voltages as high as ± 150 mV (data not shown).

The channels formed by BesC were permeable to a variety of different ions. The conductance of the 300 pS channel was found to be dependent both on the type of electrolyte and its concentration (Table 3). The KCl concentration was varied from 0.1 to 3 M and the conductance behaved as a linear function of the electrolyte concentration, meaning that the channel does not contain point charges in or near the channel mouth. Replacement of chloride against the less mobile acetate had a strong effect on the single-channel conductance, which decreased by 50% from 300 pS for 1 M KCl to 150 pS for 1 M potassium acetate. Exchange of the cation from K^+^ to Li^+^ had, in contrast to this, a relatively small effect: the single-channel conductance decreased only from 300 pS (1 M KCl) to 250 pS (1 M LiCl). This result demonstrates that the 300 pS channel shows some preference for anions over cations.

**Table 3:** Average single channel conductance, *G*, of the BesC channels in different salt solutions.

Salt	Concentration, M	Average single channel conductance, pS
LiCl	1	250
KCl	0.1	30
KCl	0.3	90
KCl	1	300
KCl	3	900
KCH_3_COO, pH 7	1	150

The membranes were formed from 1% diphytanoylphoshatidylcholine dissolved in *n*-decane. The pH of the aqueous salt solutions was 6 if not indicated otherwise. The single-channel conductance is given as the mean of at least 100 single events. The applied voltage was 20 mV and the temperature was 20°C.

### Selectivity of the BesC channel

Zero-current membrane potential experiments were performed to analyze the ion selectivity. Table 4 shows the results of measurements taken in the presence of 5-fold salt gradients of KCl, LiCl and potassium acetate. After insertion of 100 to 1000 channels into the phosphatidylcholine membrane, the salt concentration on one side of the membrane was raised from 100 to 500 mM by addition of 3 M salt solution. The aqueous phase was stirred for equilibration and 10 min after increasing the salt gradient the zero-current potential across the membrane was measured. For potassium acetate the potential was found to be positive on the more diluted side of the membrane, whereas it was found to be negative for LiCl on the same side. The zero-current membrane potential was close to zero for KCl, which means that the ion permeability through the BesC pore follows the aqueous mobility of the ions. Analysis of the zero-current membrane potential using the Goldman Hodgkin Katz equation [27] revealed a permeability *P_cation_*/*P_anion_* of 0.6 (LiCl), 0.9 (KCl) and 2.4 (potassium acetate), respectively.

**Table 4:** Zero-current membrane potential, *V*_m_ of diphytanoylphoshatidylcholine/*n*-decane membranes in the presence of BesC measured for a 10-fold gradient of different salts.

Salt	*V*_m_ a, mV	Permeability ratio *P*_cation_/*P*_anion_
KCl	− 0.1	0.9
LiCl	− 8.7	0.6
KCH_3_COO, pH 7	10	2.4

a *V*_m_ is defined as the difference between the potential at the diluted side (100 mM) and the potential at the concentrated side (500 mM). The pH of the aqueous salt solution was 6 unless otherwise indicated. *t*  =  20°C. *P*_cation_/*P*_anion_ was calculated with the Goldman-Hodgkin-Katz equation [84] from at least three individual experiments.

### A structural model explains the electrophysiological properties of BesC

To further analyze the electrophysiological properties of BesC in comparison with the best characterized channel tunnel TolC of *E. coli*, we have modeled the structure of BesC. A sequence alignment with sequences of structurally known channel tunnels [13],[28],[29] revealed that BesC shares important structural determinants of the channel tunnel family (http://npsa-pbil.ibcp.fr/) (Text S1). For example, BesC contains prolines at positions P44 and P251 that are highly conserved within the TolC protein family. These proline residues are strictly required to accommodate the abrupt turn that links the β-barrel to the α-helical motifs. Similarly, the glycine residues at positions 151 and 370 are also conserved and are situated in the turns near the closed end of the periplasmic domain. However, the main differences are found at the amino- and carboxyterminal ends of the proteins. The carboxy-terminus of BesC is the shortest. It ends directly after helix 8 and does not form an extra structure within the equatorial domain as the 19 or 66 residues of the carboxy-termini of OprM or TolC, respectively. The length of the amino-terminus outside the tunnel structure is for BesC in the same range as that of TolC (13 and 10 residues, respectively. In contrast to these the OprM amino-terminal end is 61 residues long and possess an acylation site, which anchors it in the outer membrane. There are also minor variations in the length of the extracellular loops. It is noteworthy however, that these variations as well as those of the equatorial domain also have been reported for other TolC homologues [14].

**Figure 4:**
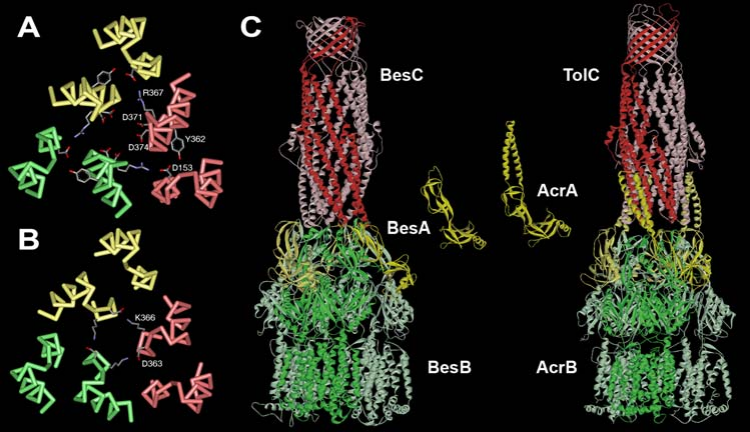
Comparison of the periplasmic tunnel entrance of *E. coli* TolC with the modeled structure of *Borrelia* BesC and a model of the *Borrelia* efflux pump. The backbone of the peptide chains of the three monomers are colored differently. Side chains are omitted except those lining the tunnel entrance. These are D371 and D374 in TolC (A) and D363 and K366 in BesC (B) or those involved in forming the circular network in TolC - D153, Y362, and R367. (C) The channel-tunnel BesC is colored red, the adaptor protein BesA is shown in yellow and the RND transporter in green. Structures of all three components are modeled and formed according to existing models [28],[82],[83]. The model of the adaptor protein BesA shows just residues 38–219 of the mature chain. The remaining parts of the protein are not modeled because of the missing template. In this model the complex is comprised of three adaptor protein protomers. It should be mentioned that there are other models proposed suggesting six adaptor protein protomers per efflux apparatus [31],[59]. Note that the adaptor proteins do not have the alpha-helical domain, which is thought to interact with the helices of the tunnel region of the outer membrane component in other bacterial efflux pumps.

To explain our biophysical data, a special focus was put on residues lining the periplasmic entrance, which are known to have a major influence on the electrophysiological properties of channel-tunnels [30]. In comparison to TolC of *E. coli*, which forms pores of 80 pS in 1 M KCl with a high preference for cations due to six aspartate residues (Asp371 and Asp374) lining the tunnel entrance (Figure 4A), the pores formed by BesC are almost non-selective. Looking at the modeled BesC tunnel entrance, it becomes apparent that the opening is slightly wider than that of TolC, explaining the higher single channel conductance. Furthermore, the charges lining the channel entrance are balanced. There are two oppositely charged residues per monomer, Asp363 and Lys366, which could explain the low ion selectivity of BesC (Figure 4B).

### The adaptor protein of the *Borrelia* multi-drug efflux pump is atypical

Sequence alignments with homologs of the other two proteins, which are part of the *Borrelia* drug efflux pump, showed no major differences for the RND transporter BesB compared to other members of the family (Text S1). There is however a striking difference in BesA compared to other adaptor proteins involved in multi-drug efflux. The sequence alignment reveals that the alpha-helical domain, which is located between the two halves of the highly conserved lipoyl domain, is missing in BesA. The loop, which connects the two halves is just nine residues long instead of the 61 or 75 residues found in the proteins MexA of *P. aeruginosa* or AcrA of *E. coli*, respectively. This means that the long coiled coil domain, which is suggested to stabilize the contact to the channel-tunnel in the assembled complex of AcrABTolC or MexABOprM, is missing [31]. Thus, in contrast to other efflux pumps investigated so far with extensive contact sites between the inner and outer membrane components, this contact is restricted to head-to-tail interactions between the periplasmic end of BesC at one site and the uppermost part of the inner membrane complex formed by BesA and BesB in the *Borrelia* efflux pump (Figure 4C).

## Discussion

In this study, we characterized a putative RND-type efflux system in *Borrelia burgdorferi*. Based on the sequence homology to other multi-drug efflux systems, we conclude that the *besB*, *besA*, and *besC* genes encode an efflux system in *B. burgdorferi*. This was further supported by analysis of BesC channel-forming activities in a black lipid bilayer assay.

β-lactam antibiotics, macrolides, and tetracyclines are generally recommended for stage-dependent therapy of Lyme borreliosis, and borreliae are resistant to aminoglycosides and quinolones, such as ciprofloxacin acid and ofloxacin [32]. Existing evidence indicates that the possible heterogeneity of *B. burgdorferi* may enable certain isolates to evade antimicrobial therapy and may account for the subsequent relapses suffered by some patients [33],[34],[35]. The mechanisms and actual proteins involved in *Borrelia* resistance to different antimicrobial agents have not been elucidated. Therefore our finding of increased *Borrelia* susceptibility to several antibiotics (Table 1) due to inactivation of BesC, the homolog of *E. coli* TolC, is the first proof that *Borrelia* possesses an active efflux system which might be responsible for multi-drug resistance. This idea can be supported by studies of *E. coli* TolC and its role in efflux of different substances including penicillins and tetracyclines [25].

The emergence of active efflux as a major causative factor in antibiotic resistance has been one of the most significant trends in anti-infective chemotherapy over the last decade and strategies to identify efflux pump inhibitors are in progress [36]. For these reasons, the identification and the characterization of such an efflux system in *B. burgdorferi* is very important for understanding the pathogenicity of this organism.

There is accumulating evidence that efflux pumps conferring clinically relevant antibiotic resistance are important for bacterial pathogenicity. This was further strengthened in a recent study by Gil and coworkers that the deletion of a TolC ortholog in *Francisella tularensis* affected its role both in virulence and in antibiotic resistance [37]. It is possible that in some species efflux pumps exporting antimicrobial agents also are important for colonization and infection of human and animal cells [38],[39]. Several studies have shown that lack of efflux-pump expression by a Gram-negative bacterium has a deleterious effect on the ability of the bacterium to be pathogenic in animal models [40],[41]. Some bacterial efflux pumps export not only antibiotics and other substances, but also host-derived antimicrobial agents [42]. This finding has led to the suggestion that the physiological role of these systems is evasion of such naturally produced molecules, thereby allowing the bacterium to survive in its ecological niche [43].

In Gram-negative bacteria, all three components of the efflux systems are often encoded within the same gene cluster, as is the case for *mexAB-oprM* from *Pseudomonas aeruginosa* [44]. However, in some efflux systems, the gene encoding the OMF component is not in the same gene cluster as the other two components. In *B. burgdorferi*, the genes coding for the three components are clustered, and form one transcriptional unit. Furthermore, in several Gram-negative bacteria, more than one TolC homologue and multiple multi-drug efflux systems are present [11]. This is not the case in *B. burgdorferi*. Here, the *besABC* system is the only multi-drug efflux pump identified thus far in the genome. In addition to the TolC efflux protein, multi-drug efflux and type I secretion systems require a periplasmic adaptor protein and an energy-providing protein [17],[45].

Functional multi-drug efflux pumps need several components. BesC needs to interact with the putative inner membrane complex formed by the AcrB homologue BesB, which serves as an inner membrane transporter, and the AcrA homologue BesA, a periplasmic adaptor protein. The genes coding for these three proteins are likely to form an operon within the chromosome of *B. burgdorferi*. These three components are necessary to form a transport system spanning the two membranes for pumping out noxious compounds from the cytoplasm [25],[46]. The special characteristic of the *Borrelia* adaptor protein is the absence of the hairpin domain. Several experiments with *E. coli* and *P. aeruginosa* efflux pumps support the idea that the hairpin domain is involved in stabilization of the contact between inner and outer membrane components [31],[47]. In the case of the *Borrelia* efflux pump, the modeled structures of the three *Borrelia* components suggest that the contact zone is restricted to the head-to-tail contact between the BesC channel-tunnel and the BesB/BesA complex. In this interaction charged residues might play a role. There are negatively charged residues on the tip of the tunnel structure and positively charged residues at loops forming the rim of the funnel structure of BesB. Also the nine-residue-long loop replacing the helical domain in BesA contains two positively charged residues, which might be involved in the interaction.

It is widely believed that the opening of the tunnel entrance is triggered by the interaction with the inner membrane complex. In the efflux pumps investigated and modeled by now it is obvious that the coiled coil domains of the adaptor proteins provide a large interface for such interaction. However, biochemical data of the *E. coli* efflux pump also provide evidence that loops of the upper rim of the inner membrane transporter AcrB are in contact with the channel tunnel and might also trigger and stabilize the tunnel opening [31],[47],[48]. It must be assumed that in the case of the Borrelia efflux pump this interaction is sufficient to trigger and stabilize opening of BesC. One might speculate that compared to the more tightly constricted periplasmic opening of OprM, which opening requires larger conformational changes and probably more energy input, possibly provided by the stronger interaction due to the coiled coil domains of the adaptor proteins, the already wider tunnel entrance of BesC requires less conformational changes and less interaction for the transition into the open state.

One can assume that the lack of the coiled coil domain of the BesA protein concomitant with a much smaller interaction site between the inner membrane complex and the outer membrane component results in a less stable assembly compared to drug efflux pumps like AcrABTolC or MexABOprM. From an evolutionary point of view the Borrelia efflux pump presents an archetypical form of this apparatus because the adaptor protein lacking the coiled coil domain resembles most the potential progenitor, the lipoyl-domain of acetyl transferase proteins [49]. This is consistent with the fact that Spirochaete present a very early branch in evolution of bacteria [50].

Separation of the outer membrane proteins from a *p66* knock out strain by anionic exchange chromatography resulted in a fraction, which showed a uniform channel-forming activity in the black lipid bilayer assay with a single-channel conductance of 300 pS in 1 M KCl. By mass spectrometry using peptide mass fingerprints [51] we identified the protein BesC as the sole protein with a putative pore-forming function. A BLAST search and a NCBI conserved domain search identified BesC as a homologue of *E. coli* TolC. TolC is the outer membrane component of type I secretion systems and multi-drug efflux pumps [45]. By itself, TolC forms a channel with a single-channel conductance of 80 pS in 1 M KCl, which is almost four-fold smaller as that for the channel in the isolated FPLC fraction [13],[45],[52],[53]. TolC is a homotrimer with a 140 Å long cannon-shaped structure and each of the three monomers contributes four β-strands to form a single 40 Å long β-barrel, the so-called channel domain, which is anchored in the outer membrane [13]. The β-barrel is extended by a 100 Å long tunnel domain formed exclusively by α-helices. The alignment of BesC with TolC of *E. coli* and OprM of *P. aeruginosa* (Text S1) reveals conserved residues, which are important for the correct folding of the channel-tunnel family. Among these are proline and glycine residues, which are important for the transitions between the β-sheets of the channel and the α-helices of the tunnel [54]. An immunoblot analysis with BesC antibodies showed that the protein is present in the FPLC fraction, which also is active in the black lipid bilayer (data not shown).

For TolC of *E. coli* it is known that residues lining the periplasmic entrance are critical for the electrophysiological behavior. The aspartate ring consisting of six aspartates residues at the TolC tunnel entrance from the periplasm is responsible for its high cation selectivity [55]. In BesC this aspartate ring is replaced by positive lysine residues (K366) and negative aspartate residues (D363) resulting in a non-selective BesC channel, in contrast to the cation selective TolC channel. The lysine residue (K366) and the aspartate residue (D363) could neutralize one another, meaning that no net charge exists in the tunnel entrance resulting in a non-selective channel. Another difference between BesC and TolC is that the BesC channel has a nearly four-fold higher conductance in 1 M KCl than TolC (80 pS in 1 M KCl) [52]. TolC of *E. coli* contains a circular network of inter- and intra-molecular connections between the three monomers that is responsible for the stability of the almost closed state of the channel-tunnel [56]. This network involves residues located at the inner and outer coiled coil of the tunnel domain, which are not conserved in BesC (see Figure 4A). This suggests that other interactions keep the helices of the tunnel entrance together; otherwise the single-channel conductance would be much higher. The modeled BesC structure shows that the two charged residues D363 and K366 of different monomers are in close proximity (2.1 Å), allowing formation of intermolecular salt bridges. Thus, they are able to establish a similar circular network keeping the helices at the tunnel entrance in a close conformation.

The existence of a multi-drug efflux system in *B. burgdorferi* makes sense because *B. burgdorferi* carries natural resistance for several antibiotics [57]. Resistance can originate from multi-drug efflux pumps, as has previously been demonstrated for several Gram-negative bacteria [25],[58],[59],[60].

In this article we described three genes, *besB*, *besC* and *besA*, which encode proteins comprising a multi-drug efflux system in *B. burgdorferi*. We further showed that the BesC protein, a TolC homolog, is a novel virulence factor of *B. burgdorferi*. This efflux system might be part of a Type I secretion machinery for maintenance of cellular homeostasis or export of exogenous toxic agents, perhaps necessary for survival in vastly different host environments. The finding of this novel system in borreliae offers the potential to develop anti-borrelial therapeutics by screening compounds affecting the efflux system. Further functional studies will open new possibilities to investigate and understand the virulence mechanisms of *Borrelia* spirochetes.

## Materials and Methods

### Bacterial strains and growth conditions

Infectious, low-passage strain B31 [61] was used for RNA extraction and operon analysis. Strain *p66::str* [26] containing P_flaB_-*aadA* insertion in the *p66* gene was used for constructing *besC* mutant (*p66::str*/*besC::kan*) used in planar lipid bilayer assay. For animal infectivity studies strain 5A4NP1 [62], infectious clone of B31 containing a disruption of nicotinamidase gene *pncA* (*bbe22*) carried on plasmid lp25, was used to create *besC* knock-out and complementation strains, respectively, *besC::str* and *besC::str* + *besC*. Unless otherwise stated, bacteria were grown in BSK-II medium [63] supplemented with 6% rabbit serum (Sigma) at 35°C until the cell density reached approximately 10^7^–10^8^ cells ml^−1^. *E. coli* Top10 (Invitrogen), used for cloning experiments and *E. coli* ROSETTA (Novagen), used for protein overexpression, were grown at 37°C in Luria-Bertani (LB) broth or on LB agar plates, containing 50 µg/ml of carbenicillin, 5 µg/ml gentamicin or 50 µg/ml kanamycin when needed.

### Sequence analysis and protein modeling

The amino acid sequences of BesC (BB0142), BesA (BB0141) and BesB (BB0140) were analyzed using BLAST and the Conserved Domain Database at the National Center for Biotechnology Information (http://www.ncbi.nlm.nih.gov/). Homologous sequences were aligned using the ClustalW program (http://www.ebi.ac.uk/clustalw/). Based on sequence alignments, protein modeling was performed on the swiss-model platform (http://swissmodel.expasy.org/; Text S1). For modeling of BesC (BB0142) the coordinates of OprM (pdb: 1WP1), for BesA (BB0141) the coordinates of MexA (pdb: 1T5E) and for BesB (BB0140) the coordinates of AcrB (pdb: 2HRT) were used.

### Construction of *besC* gene inactivation and complementation plasmids

Plasmid pOK-*besC::kan* was constructed to inactivate the *besC* gene in *B. burgdorferi p66::str* strain. Primers besC-XhoI-f and besC-BamHI-r (Table S1) containing *Xho*I and *Bam*HI restriction sites were used to amplify a fragment of 2189 bp, covering the *besC* gene, 426 bp upstream and 440 bp downstream from the gene. Purified PCR product was ligated into *Bam*HI- and *Xbo*I-digested pOK12, a 2.1-kb low-copy-number plasmid containing a kanamycin resistance gene [64]. By using primers besC-NcoI-f and besC-PstI-r (Table S1) containing *Nco*I and *Pst*I restriction sites the whole plasmid was amplified and a 1.3-kb P*_flaB_*-kanamycin fragment described elsewhere [65] was amplified with primers kan-F-PstI and kan-R-NcoI. The *kan* fragment was then subcloned into the *Pst*I- and *Nco*I-digested amplicon of the *besC* gene and pOK12 vector. A similar strategy was applied to construct the plasmid pOK-*besC::str* which was used to inactivate *besC* gene in *B. burgdorferi* 5A4NP1 strain. Except for selection marker, a 1.3-kb P*_flgB_*-streptomycin fragment [66] was amplified using primers aada-F-PstI and aada-R-NcoI and subcloned into the *Pst*I- and *Nco*I-digested amplicon of *besC* gene and pOK12 vector. Complementation plasmid pCOMP was constructed based on pBSV2G shuttle vector [67]. Primers besABC-BamHI and besABC-PstI (Table S1) containing *Bam*HI and *Pst*I restriction sites were used to amplify a 6299 bp fragment, covering the *besC*, *besA*, *besB* genes with an additional 425 bp upstream and 457 bp downstream of the genes. This product was subjected to restriction enzyme digestion and ligated to *Bam*HI- and *Pst*I-digested pBSV2G shuttle vector.

### Electroporation of *B. burgdorferi* and screening of transformants

Preparation of competent *B. burgdorferi* cells and electroporation was done as described previously [68],[69]. Single clones were obtained, as described elsewhere [70], in the presence of the appropriate selective antibiotics: streptomycin (50 µg ml), gentamycin (40 µg ml), and/or kanamycin (200 µg ml). Transformants were further analyzed using primers described in Table S1.

### Determination of the plasmid profile and recovery of shuttle vector

Total genomic DNA from *B. burgdorferi* strains, was prepared using a Wizard genomic DNA purification kit (Promega). Plasmid contents of the *B. burgdorferi* strains 5A4NP1, *besC* mutant, and complemented *besC* mutant were determined by PCR as previously described by Elias *et al.* [71]. The *B. burgdorferi* strains recovered from the mouse organs were also tested for plasmid content as described above. To determine the presence of the complementation plasmid in the complemented *besC* mutant recovered from mouse organs, total DNA from this strain was transformed into *E. coli* cells. Gentamycin-resistant clones were screened by PCR for the presence of the shuttle vector.

### Separation of the outer membrane proteins of *B. burgdorferi* and purification of BesC

Outer membrane proteins (B-fractions) of *B. burgdorferi* strains *p66::str* (a clone with an inactivated *p66* gene) and *p66::str/besC::kan* (a clone with inactivated *besC* and *p66* genes) were prepared by detergent (octyl-glucopyranoside) extraction as described elsewhere [72]. Purification of native BesC was performed by anion exchange MonoQ chromatography in combination with fast protein liquid chromatography (FPLC) (Amersham Biosciences). About 200 µg of *p66::str* fraction B was dissolved in 800 µl 2% lauryl-dimethyl-amine-oxide (LDAO, Sigma) and applied to the column. The column was first washed with 7.5 ml 0.4% (LDAO) buffered with 10 mM Tris-HCl (pH 8.0). Bound proteins were eluted with a linear NaCl gradient (0 to 1 M) containing 0.4% LDAO buffered with 10 mM Tris-HCl (pH 8.0). Fractions showing a peak in the FPLC chromatogram were further analyzed by SDS-PAGE and black lipid bilayer assay. A control FPLC was performed as described above with about 200 µg of *p66::str/besC::kan* fraction B.

### Overexpression of recombinant constructs of BesC, BesA, and BesB

For overproduction of BesC, BesA, and BesB fragments in *E. coli* ROSETTA, the pET-M11 plasmid [73] was used. The gene fragments were amplified by PCR by using oligonucleotides described in Table S1. After restriction enzyme digestion, the PCR products were ligated into the plasmid pET-M11. The *E. coli* cells carrying expression plasmids were grown at 37°C to OD_600_  =  0.6 in LB medium containing 50 µg of kanamycin per ml and protein expression was induced by addition of isopropyl-β-d-thiogalactopyranoside (IPTG) to a final concentration of 1 mM. The culture was incubated further for 2 h, and cells were collected by centrifugation at 6,000 × *g* for 15 min. The cells were suspended in 0.1 culture volume of 20 mM Tris-HCl (pH 8.0). Lysozyme (Sigma) was added to a final concentration of 0.1 mg/ml, and the cells were disrupted by sonication. The soluble and insoluble fractions were separated by centrifugation at 10,000 × *g* for 15 min. The recombinant protein was expressed in inclusion bodies. Inclusion bodies were washed twice with 40 ml of wash buffer (20 mM Tris-HCl [pH 7.5], 10 mM EDTA, 1% Triton X-100) and used to raise antiserum against *B. burgdorferi* BesC, BesA, and BesB.

### Antibodies

Polyclonal antiserum was raised against recombinant fragments of BesC, BesA, and BesB produced as described above. One milligram of washed inclusion bodies was separated on a sodium dodecyl sulfate (SDS)—12.5% polyacrylamide gel electrophoresis (PAGE) gel. Recombinant protein was excised from the gel and approximately 100 µg of protein was used for rabbit immunization and subsequent boosts. As a control, murine monoclonal antibody H9724 [74], which recognizes the constitutively expressed FlaB (flagellin) protein was used.

### Protein electrophoresis, immunoblotting, and antiserum

Total *B. burgdorferi* proteins were prepared from cells grown to stationary phase by harvesting the cells by centrifugation, and washing twice in phosphate-buffered saline. For gel electrophoresis, proteins were boiled for 5 min in NuPAGE sample buffer and separated through 4 to 12% NuPAGE bis-Tris polyacrylamide gels (Invitrogen). For immunoblotting, proteins were transferred to a polyvinylidene difluoride membrane (PVDF) (PALL Corporation) and probed with antibodies. Bound antibodies were detected using peroxidase-conjugated anti-rabbit or anti-mouse antibodies (DAKO A/S) and enhanced chemiluminescence reagents according to the manufacturer's instructions (Amersham Pharmacia Biotech).

### Planar lipid bilayer assay

The methods used for the black lipid bilayer experiments have been described previously [75]. For preparation of the artificial lipid membranes, a 1% solution of diphytanoyl phosphatidylcholine (Avanti Polar Lipids) in *n*-decane was used. All salts (analytical grade) were purchased from Merck. The aqueous salt solutions were used without buffering and had a pH around 6 unless otherwise indicated. The temperature was kept at 20°C throughout. The channel-forming protein solutions were diluted in 1% Genapol X-080 (Fluka) and added to the aqueous phase after the membrane turned black. The membrane current was measured with a pair of calomel electrodes switched in series with a voltage source and an electrometer (Keithley 617). For single-channel recordings the electrometer was replaced by a highly sensitive current amplifier (Keithley 427). The amplified signal was recorded with a strip chart recorder. The zero-current membrane potentials were measured as described previously [27]. The membranes were formed in a 100 mM salt solution containing a predetermined protein concentration so that the membrane conductance increased about 100–1000 fold within 10–20 min after membrane formation. At this time the instrumentation was switched to the measurements of the zero-current potential and the salt concentration on one side of the membrane was raised by adding small amounts of concentrated salt solutions. The zero-current membrane potential reached its final value after 10 min. The voltage dependence of the porin channel was checked as described elsewhere [76] using membrane potentials as high as −150 to +150 mV.

### Mass spectrometry

The FPLC fractions showing channel-forming activity were subjected to SDS-PAGE followed by silver staining [77]. The different bands were analyzed by nano LC-mass spectrometry as described elsewhere [78]. Data interpretation of the MS/MS datasets was performed by the Mascot algorithm [79]. In detail an Ultimate II (LC Packings, Germering, Germany) in combination with an ESI – iontrap (LCQ Deca XP, Thermo, Dreieich, Germany) were used. Mass spectra obtained by LC-MS/MS analysis were used to identify the corresponding peptides with the MascotTM [79] (version 2.1.6). The algorithm searched in the NCBI database (16.08.2005) restricted to the bacteria protein taxonomy with the following parameter set: (a) fixed modification: carbamidomethyl (C); (b) variable modification: oxidation (M); (c) peptide and MS/MS tolerance: +/− 1.5 Da; (d) ion score cut-off: 30.

### Microdilution susceptibility testing, determination of MIC and MBC values

Compounds tested belonged to classes of penicillins (penicillin G, carbenicillin; Sigma-Aldrich), tetracyclines (tetracycline; Sigma-Aldrich), macrolides (azithromycin; Sigma-Aldrich), cephalosporins (cefotaxime; Sigma-Aldrich), detergents (sodium dodecyl sulfate; Scharlau Chemie) and intercalators (ethidium bromide; Bio-Rad). To test different concentrations, serial dilutions of substances were done in 96-well Microtest plates (Falcon). A colorimetric assay was used for susceptibility testing as described elsewhere [80],[81]. Briefly, *B. burgdorferi* 5A4NP1, *besC::str* and the complemented strain were cultured in BSK-II [63] at 35°C to log phase and adjusted to 5×10^7^ bacteria per ml as determined by enumeration with a Petroff-Hausser bacteria counting chamber (C.A. Hausser & Son). Microtiter wells were seeded with 5×10^6^ bacteria and antibiotic in a final volume of 200 µl. The ranges of concentrations tested were as follows (µg/ml): penicillin G 0.001–20; carbenicillin 0.00015–5; tetracycline 0.00035–10, azithromycin 0.00006–2; cefotaxime 0.001–25; ethidium bromide 0.002–50 and sodium dodecyl sulfate 1.5–50000. Microtiter trays with *Borrelia* samples and growth controls were covered with a low evaporation lid supplied by the plate manufacturer (Falcon) and cultured at 35°C with 1% CO_2_. Growth was examined after 0, 24, 48, and 72 h by measurement of indicator color shift at 562/630 nm using an ELISA reader (Multiscan RC, Labsystems) in combination with a software-assisted calculation program (Genesis Lite 3.03, Life Sciences Ltd).

Colorimetric MIC's of isolates were measured in triplicate by quantification of growth utilizing a software-assisted calculation (Genesis Lite 3.03, Life Sciences Ltd.). Growth of samples was determined for each well based on the decrease of absorbance after 72 h (Et_72_) in comparison to the initial absorbance values (Et_o_). The lowest concentration of antibiotic at which no color shift could be detected was interpreted as the MIC.

Minimal borreliacidal concentration (MBC) values for tested compounds were determined in following way. Aliquots (50 µl) from all vials without demonstrable growth were inoculated into 5 ml of fresh BSK medium (dilution factor 1:100) to achieve a sample dilution below the MIC and then incubated at 35°C at 1% CO_2_ for an additional 3 weeks. After gentle agitation of the subcultures, 5–10 high power fields were examined by dark-field microscopy for the presence or absence of viable *Borrelia*. The MBC was defined as the lowest concentration of the antimicrobial agent where no spirochetes could be detected after three weeks of subculture.

For each strain and substance three independent experiments were performed on different days.

### Isolation of RNA and RT-PCR

All reagents were prepared with diethylpyrocarbonate (DEPC)-treated water. Total RNA was isolated from *in vitro*-cultured *Borrelia* using the Ultraspec-II RNA isolation system (Biotex Laboratories) according to the manufacturer's instructions. RNA was quantified using a NanoDrop spectrophotometer (NanoDrop Technologies). To remove contaminating genomic DNA, RNA samples were treated with 3 units of RNase-free DNaseI (Roche). For RT-PCR the Superscript One-Step RT-PCR with Platinum Taq kit (Invitrogen) was used following the manufacturer's instructions. A negative control with sterile water was used to verify the purity of the reagents. The absence of DNA contamination was verified by PCR. Ten microliters of each RT-PCR product was analyzed on a 1% agarose gel stained with ethidium bromide (5 µg ml^−1^). The primers b1 and a1 were used to detect a transcript spanning *besB (bb0140)* and *besA (bb0141)*, the primers a2 and c1 were used to amplify a transcript spanning *besA (bb0141)* and *besC (bb0142)*, the primers b1 and c1 were used to detect a transcript spanning *besB (bb0140)* and *besC (bb0142)*. Primer sequences are shown in Table S1.

### Animal infectivity studies

*B. burgdorferi* 5A4NP1 infectious strain, *besC::str* mutant strain and *besC::str* + *besC* complemented strain were used for mouse infections. Seven four-week-old C3H/HeN mice (Bomholt Gård Breeding) per strain were subcutaneously injected with 10^6^ spirochetes in 0.1 ml culture medium. The number of bacteria was determined microscopically in a Petroff–Hausser chamber. After 2 weeks mice were sacrificed and heart, bladder, knee, and ear were removed from each mouse and incubated for 4 weeks in BSK-II medium containing 7% rabbit serum and supplemented with sulfamethoxazole (1.25 µl ml^−1^) and phosphomycin (4 µl ml^−1^). Each sample was examined for the presence of spirochetes by dark field microscopy.

### Accession numbers

The GenBank (http://www.ncbi.nlm.nih.gov/Genbank/index.html) accession numbers of guinea pig genes used for primer design are as follows: β-actin (AF508792.1); IFNγ (AY151287.1); IL-1β (AF119622); MCP-1 (L04985); MCP-3 (AB014340); RANTES (CPU77037); TLR3 (DQ415679.1); and TNFα (CPU77036).

The Protein Data Bank (http://www.rcsb.org/pdb/) ID numbers for the structures discussed in this paper are crystal form 1 (2Q8A) and crystal form 2 (2Q8B).

## Supporting Information

Figure S1 Outer membrane proteins of *B. burgdorferi* B31-A Δ*p66::str* were separated by anion exchange chromatography. Fractions showing uniform channel-forming activity with a single-channel conductance of about 300 pS were precipitated, separated by SDS-PAGE and silver stained.
(0.07 MB DOC)

Table S1 Oligonucleotide primers used in this study.
(0.06 MB DOC)

Table S2 Identification of BesC (BB0142) by peptide mass fingerprinting.
(0.07 MB DOC)

Text S1 Sequence alignment
(0.08 MB DOC)
