# Supplementary material for: An RND-Type Efflux System in Borrelia burgdorferi Is Involved in Virulence and Resistance to Antimicrobial Compounds
Source: PLoS Pathog. 2008 Feb 29;4(2):e1000009. doi: 10.1371/journal.ppat.1000009 (PMC2279261; doi:10.1371/journal.ppat.1000009)

**Figure S1.** Outer membrane proteins of *B. burgdorferi* B31-A *∆p66::str* were separated by anion exchange chromatography. Fractions showing uniform channel-forming activity with a single-channel conductance of about 300 pS were precipitated, separated by SDS-PAGE and silver stained.


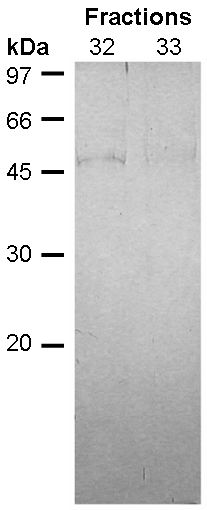

Supplement: Figure S1 — Outer membrane proteins of B. burgdorferi B31-A Δp66::str were separated by anion exchange chromatography. Fractions showing uniform channel-forming activity with a single-channel conductance of about 300 pS were precipitated, separated by SDS-PAGE and silver stained. (0.07 MB DOC) [file ppat.1000009.s001.doc]
