# Supplementary material for: An RND-Type Efflux System in Borrelia burgdorferi Is Involved in Virulence and Resistance to Antimicrobial Compounds
Source: PLoS Pathog. 2008 Feb 29;4(2):e1000009. doi: 10.1371/journal.ppat.1000009 (PMC2279261; doi:10.1371/journal.ppat.1000009)
Supplement: Table S1 — Oligonucleotide primers used in this study. (0.06 MB DOC) [file ppat.1000009.s002.doc]

**Table S1.** Oligonucleotide primers used in this study.

| **Name** | **5’-3’ nucleotide sequence ^a^** | **Application** |
| --- | --- | --- |
| besB-ab-NcoI-f | CGTTT**CCATGG**AATTTTATCATGGAACCGATTTAG | Overexpression |
| besB-ab-KpnI-r | CGTTTT**GGTACC**GATTAAGAAATTGAAGGCAAC | Overexpression |
| besA-ab-NcoI-f | TATTG**CCATGG**TTTCTAAAATCAAACTTATTACTAAGC | Overexpression |
| besA-ab-KpnI-r | ATCCC**GGTACC**AATTAAATATTGCTTTCGGCTGAA | Overexpression |
| besC-ab-NcoI-f | ATTAT**CCATGG**GATTAGATCATGATCAAGATTTTG | Overexpression |
| besC-ab-KpnI-r | CATCAA**GGTACC**TCTTAGTCTAATGAATTTATTAAAT | Overexpression |
| besC-XhoI-f | TATGTTGGA**CTCGAG**ATTAACTGTTTCACC | Gene inactivation |
| besC-BamHI-r | ATAA**GGATCC**AGGCAAAGAATACCGCAATGC | Gene inactivation |
| besC-NcoI-f | CAAGCTCAC**CCATGG**CAGAAGGATTTCTAC | Gene inactivation |
| besC-PstI-r | CAAATGGCATTTA**CTGCAG**TTAATTCCGGGGTC | Gene inactivation |
| aada-F-PstI | ACGGC**CTGCAG**TGTGCTGGAATTC | Gene inactivation |
| aada-R-NcoI | CCAGTG**CCATGG**ATATCTGCAGAATTC | Gene inactivation |
| kan-F-PstI | TAAAA**CTGCAG**CCGGCTGTCTGTC | Gene inactivation |
| kan-R-NcoI | CTCTG**CCATGG**TTACAACCAATTAACC | Gene inactivation |
| besABC-BamHI | CCACTCCCTT**GGATCC**CAAAACAGC | Complementation |
| besABC-PstI | TATGTTGGA**CTGCAG**ATTAACTGTTTCACC | Complementation |
| besC-m1 | TTAATTTTAGAAGCATCAAGAATCGC | Mutant analysis |
| besC-m2 | GGATTAATCTTTCTTTGTCCCCTTC | Mutant analysis |
| besA-m1 | CAACCTCAATAATAGCATCATTTCC | Mutant analysis |
| aadA-1 | GAAGCGGTGATCGCCGAAG | Mutant analysis |
| aadA-2 | AACTGATCTGCGCGCGAG | Mutant analysis |
| pBSV-r | GACGTTGTAAAACGACGGCCAG | Mutant analysis |
| b1 | CTTGGGGTCTGTGATTTGCTGG | Operon analysis |
| a1 | CGGGCGAGGTTGAAGAGAATG | Operon analysis |
| a2 | ATTGGCGCTCTTACAGGACTTTTC | Operon analysis |
| c1 | TTCCGGGGTCATGGATCTTTCT | Operon analysis |
| c2 | GATCCATGACCCCGGAATTAAAAG | Operon analysis |
| c3 | GGAGAATTGCCAGACGAAACAATAG | Operon analysis |

^a^ Restriction sites are indicated by boldface type
