# Supplementary material for: An RND-Type Efflux System in Borrelia burgdorferi Is Involved in Virulence and Resistance to Antimicrobial Compounds
Source: PLoS Pathog. 2008 Feb 29;4(2):e1000009. doi: 10.1371/journal.ppat.1000009 (PMC2279261; doi:10.1371/journal.ppat.1000009)
Supplement: Table S2 — Identification of BesC (BB0142) by peptide mass fingerprinting. (0.07 MB DOC) [file ppat.1000009.s003.doc]

**Table S2.** Identification of BesC (BB0142) by peptide mass fingerprinting.

| **General** |  |  | **MS** |  |  |  |  |  |  |  |  |  |  |
| --- | --- | --- | --- | --- | --- | --- | --- | --- | --- | --- | --- | --- | --- |
| accession number | protein name | MW | cummulative score | sequence coverage [%] | number of single peptides | peptide charge | observed mass | Mr(expt) | Mr(calc) | delta | miss | score | peptide |
| NP_212276 | BB0142 | 50815 | 127 | 19 | 8 | 2 | 525.57 | 1049.12 | 1048.59 | 0.53 | 0 | 38 | K.SYNQLIALK.S |
|  |  |  |  |  |  | 2 | 554.14 | 1106.26 | 1105.60 | 0.66 | 0 | 44 | K.LNDIELVYK.Q |
|  |  |  |  |  |  | 2 | 558.53 | 1115.05 | 1114.56 | 0.48 | 0 | 51 | R.NPSALSELER.D |
|  |  |  |  |  |  | 2 | 664.67 | 1327.33 | 1326.68 | 0.65 | 0 | 53 | K.LNYANSILEYK.N |
|  |  |  |  |  |  | 2 | 721.19 | 1440.37 | 1440.70 | -0.33 | 0 | 67 | R.MQLVMLEYESAK.I |
|  |  |  |  |  |  | 2 | 807.13 | 1612.25 | 1611.79 | 0.46 | 0 | 54 | K.SQPDLDGHIINFEK.S |
|  |  |  |  |  |  | 3 | 538.60 | 1612.77 | 1612.80 | -0.03 | 1 | 15 | K.RMQLVMLEYESAK.I |
|  |  |  |  |  |  | 2 | 1055.63 | 2109.24 | 2109.00 | 0.24 | 0 | 64 | K.QAVNMALENSLDSENALYK.E |
|  |  |  |  |  |  |  |  |  |  |  |  |  |  |
|  |  |  |  |  |  |  |  |  |  |  |  |  |  |
| accession number: | NCBI |  |  |  |  |  |  |  |  |  |  |  |  |
| cummulative score: | cummulative score considering each single peptide score | | | | | |  |  |  |  |  |  |  |
| sequence coverage: | percentage of covered protein sequence by identified peptides | | | | | |  |  |  |  |  |  |  |
| miss: | misscleavage of trypsin | | |  |  |  |  |  |  |  |  |  |  |
| score: | significance threshold of peptide | | |  |  |  |  |  |  |  |  |  |  |
