# Supplementary material for: An RND-Type Efflux System in Borrelia burgdorferi Is Involved in Virulence and Resistance to Antimicrobial Compounds
Source: PLoS Pathog. 2008 Feb 29;4(2):e1000009. doi: 10.1371/journal.ppat.1000009 (PMC2279261; doi:10.1371/journal.ppat.1000009)
Supplement: Text S1 — Sequence alignment (0.08 MB DOC) [file ppat.1000009.s004.doc]

**Alignment of the channel tunnel proteins TolC of *E. coli*, OprM of *P. aeruginosa* and BesC (BB0142).** The model of the BB0142 structure is based on this alignment. The secondary structure elements are shown on top based on the annotation of TolC structure. SS stands for signal sequence, H for helix and S for strand. Prediction of the cellular localization by Psort reveals for BesC a score of 9.92 for outer membrane localization (cytoplasm 0.01, cytoplasmic membrane 0.01, periplasm 0.02, extracellular 0.02), which is consistent with a high probability score for the presence of a signal sequence cleaved between position 37 and 38 as predicted by SignalP (http://www.cbs.dtu.dk/services/SignalP/).

10 20 30 40 50 60 70 80

| | | | | | | |

SS | H1 |

TolC -----------------------------------------*MKKLLPILIGLSLSGFSSLSQA*ENLMQVYQQARLSNPEL

BesC --------------------*MKILIKFYNFKVLIFIIRGLQIKKIFLIFITVSFS-FA*EIIQISP-KQAVNMALENS-LD

OprM *MKRSFLSLAVAAVVLSG*CSLIPDYQRPEAPVAAAYPQGQAYGQNTGAAAVPAADIGWREFFRDPQLQQLIGVALENNRDL

SS | | H1 |

90 100 110 120 130 140 150 160

| | | | | | | |

H2 | S1 L1 S2 | H3

TolC RKSAADRDAAFEKINEARSPLLPQLGLGADYTYSNGYRDANG----INSNATSASLQLT--QSIFDMSKWRALTLQEKAA

BesC SENALYKENIKKLYKNNAWNV-FVPNVNLSSTLSRNPSALSE----LERDYWGFGFGVG-INLSLSPSVLKRMQLVMLEY

OprM RVAALNVEAFRAQYRIQRADLFPRIGVDGSGTRQRLPGDLSTTGSPAISSQYGVTLGTTAWELDLFGRLRSLRDQALEQY

H2 | | H3

170 180 190 200 210 220 230 240

| | | | | | | |

| T1 | H4

TolC GIQDVTYQTDQQTLILNTATAYFNVLNAIDVLSYTQAQKEAIYRQLDQTTQRFNVGLVAITDVQNARAQYDTVLANEVTA

BesC ESAKIERESAVRNIKLNVLKSYNQLIALKSTLKVFESQIQNSKLKFEQARIAYNNGLISEIDFLDAQLKYKKSQPDLDGH

OprM LATEQAQRSAQTTLVASVATAYLTLKADQAQLQLTKDTLGTYQKSFDLTQRSYDVGVASALDLRQAQTAVEGARATLAQY

| T1 | H4

250 260 270 280 290 300 310 320

| | | | | | | |

| | H7 | S3

TolC RNNLDNAVEQLRQITGNYYPELAALNVENFKTDKPQPVNALLKEAEKRNLSLLQARLSQDLAREQI-RQAQDGHLPTLDL

BesC IINFEKSKEIFKLLIGLDHDQ-DFEIIGELPDETIDFSLFNEALNFNESLEIKDLNMRLKMTEQLIDSLWLDTYLPSLSL

OprM TRLVAQDQNALVLLLGSGIPANLPQGLGLDQTLLTEVPAGLPSDLLQRRPDILEAEHQLMAANASIGAARAAFF-PSISL

| H5 | | H6 | S3

330 340 350 360 370 380 390 400

| | | | | | | |

L2 S4 | H8

TolC TASTGISDTSYSGSKTRGAAGTQYDDSNMGQNKVGLSFSLPIYQGGMVNSQVKQAQYNFVGASEQLESAHRSVVQTVRSS

BesC SFSYSPYKSFH--------ENSKGFSTGFLASFSLNYGLTEIFPFSKSFTKIQDNNYQLKILQNNVEGKIRNLKSSIVQK

OprM TANAGTMSRQ----------LSGLFDAGSGSWLFQPSINLPIFTAGSLRASLDYAKIQKDINVAQYEKAIQTAFQEVADG

L2 S4 | H7

410 420 430 440 450 460 470 480

| | | | | | | |

| T2 | H9 | H10

TolC FNNINASISSINAYKQAVVSAQSSLDAMEAGYSVGTRTIVDVLDATTTLYNAKQELANARYNYLINQLN-IKSALGTLNE

BesC RKDIRRYKAILDASKINVELANKNYQMAFNAFNSGVMDLSKLNDIELVYKQSDLKFIEDKLNYANSILE-YKNLINSLD-

OprM LAARGTFTEQLQAQRDLVKASDEYYQLADKRYRTGVDNYLTLLDAQRSLFTAQQQLITDRLNQLTSEVNLYKALGGGWNQ

| T2 | H9 |

490 500 510 520 530 540

| | | | | |

TolC QDLLALNNALSKPVSTNPENVAPQTPEQNAIADGYAPDSPAPVVQQTSARTTTSNGHNPFRN

BesC --------------------------------------------------------------

OprM QTVTQQQTAKKEDPQA----------------------------------------------

**Alignment of the adaptor proteins AcrA of *E. coli*, MexA of *P. aeruginosa* and BesA (BB0141).** The model of the BB0142 structure is based on this alignment. The secondary structure elements are shown on top based on the annotation of MexA structure. SS stands for signal sequence, H for helix and S for strand. Prediction of the cellular localization by Psort reveals for BesA no reasonable result (for each localization the score is 2.0). However, there is a sufficient probability for BesA is an inner membrane lipoprotein predicted by LipoP (http://www.cbs.dtu.dk/services/LipoP/), which predicts the cleavage site to be between position 25 and 26.

10 20 30 40 50 60 70 80

| | | | | | | |

SS | | S1 | | S2|

AcrA -------MNKNRGFTPLAVVLMLSGSLALTGCDDKQAQQGGQQMPAVGVVTVKTEPLQITT--ELPGRTSAYRIAEVRPQ

MexA -------MQRTPAMRVLVPALLVAIS-ALSGCGKSEAPPPAQT-PEVGIVTLEAQTVTLNT--ELPGRTNAFRIAEVRPQ

BesA MMNLIFNINLYLKKYFLVLFLVLVACVGDNKLDDKNIDKEKESSYRFPVIAMKVKKGILSDYLSLNGDVDTKVKADIFPD

SS |

90 100 110 120 130 140 150 160

| | | | | | | |

| S3 | S4 |S5 | | H1 |T1 | H2

AcrA VSGIILKRNFKEGSDIEAGVSLYQIDPATYQATYDSAKGDLAKAQAAANIAQLTVNRYQKLLGTQYISKQEYDQALADAQ

MexA VNGIILKRLFKEGSDVKAGQQLYQIDPATYEADYQSAQANLASTQEQAQ-------RYKLLVADQAVSKQQY-------A

BesA AVGKITSLRIKLGAYVQKGQIVATLDPSR---------------------------------------------------

170 180 190 200 210 220 230 240

| | | | | | | |

| |S6| |S7| |S8| |S9 | | S10 | | H3 |

AcrA QANAAVTAAKAAVETARINLAYTKVTSPISGRIGKSNVTEGALVQNGQATALATVQQLDPIYVDVTQSSNDFLRLKQELA

MexA DANAAYLQSKAAVEQARINLRYTKVLSPISGRIGRSAVTEGALVTNGQANAMATVQQLDPIYVDVTQPSTALLRLRRELA

BesA --------------PGSVYLK-SPVRAPISGYILNITKKIGETV-NPQSN-IAVVGRIDTKQILTYV---------SEKY

250 260 270 280 290 300 310 320

| | | | | | | |

|S11| | S12 | | S13 | | S14 | |S15 |

AcrA NGTLKQE-NGKAKVSLITSDGIKFPQDGTLEFSDVTVDQTTGSITLRAIFPNPDHTLLPGMFVRARLEEGLNPNAILVPQ

MexA SGQLERAGDNAAKVSLKLEDGSQYPLEGRLEFSEVSVDEGTGSVTIRAVFPNPNNELLPGMFVHAQLQEGVKQKAILAPQ

BesA ISNIKVGNDAIIEVGAYSNEKFKAKVSEISPILDSK--SRTIEVYLTPIGSNLD-KLIIGMFSKIKLITKRFKDVIKISR

330 340 350 360 370 380 390 400

| | | | | | | |

AcrA QGVTRTPRGDATVLVVGADDKVETRPIVASQAIGDKWLVTEGLKAGDRVVISGLQKVRPGVQVKAQEVTADNNQQAASGA

MexA QGVTRDLKGQATALVVNAQNKVELRVIKADRVIGDKWLVTEGLNAGDKIITEGLQFVQPGVEVKTVPAKNVASAQKADAA

BesA EAVVEREGKKFVFKVDLESKSVQMLPITVLFEIDNIVALSGEVEENDLIVVEGMSALSNGSLINLVDTKEGLSAESNI--

AcrA QPEQSKS-

MexA PAKTDSKG

BesA --------

**Alignment of the RND transporter AcrB of *E. coli*, MexB of *P. aeruginosa* and BesB (BB0140).** The model of the BB0140 structure is based on this alignment. The secondary structure elements are shown on top based on the annotation of AcrB structure. SS stands for signal sequence, H for helix and S for strand. Prediction of the cellular localization by Psort is unambiguous. The score reaches 10.0 for localization in the cytoplasmic membrane.

10 20 30 40 50 60 70 80

| | | | | | | |

| H1 |

AcrBEc -MPNFFIDRPIFAWVIAIIIMLAGGLAILKLPVAQYPTIAPPAVTISASYPGADAKTVQDTVTQVIEQNMNGIDNLMYMS

MexBPa -MSKFFIDRPIFAWVIALVIMLAGGLSILSLPVNQYPAIAPPAIAVQVSYPGASAETVQDTVVQVIEQQMNGIDNLRYIS

BesB MLVKRIVGKPITMLILFSLLLMISLYTFSRLKVDLLPGIDIPQISIHTVYPGASPREVEESVSRVLESGLSSVKNLKNIY

90 100 110 120 130 140 150 160

| | | | | | | |

AcrBEc SNSDSTGTVQITLTFESGTDADIAQVQVQNKLQLAMPLLPQEVQQQGVSIEKSSSSFLMVVGVINTDGTMTQEDISDYVA

MexBPa SESNSDGSMTITVTFEQGTDPDIAQVQVQNKLQLATPLLPQEVQRQGIRVTKAVKNFLMVVGVVSTDGSMTKEDLSNYIV

BesB SVSSKESS-TVSLEFYHGTDLDLVLNEIRDALELVKSSLPSKSQTPRI-FRYNLKNIPVMEIVINSVRPVS--ELKRYAD

170 180 190 200 210 220 230 240

| | | | | | | |

AcrBEc ANMKDAISRTSGVGDVQLFG-SQYAMRIWMNPNELNKFQLTPVDVITAIKAQNAQVAAGQL---GGTPPVKGQQLNASII

MexBPa SNIQDPLSRTKGVGDFQVFG-SQYSMRIWLDPAKLNSYQLTPGDVSSAIQAQNVQISSGQL---GGLPAVKGQQLNATII

BesB EIIKPGLERLDGVAIVTVNGGSKKRVLIEVSQNRLESYGLSLSRISSIIASQNLELSAGNILENNLEYLVEVSGKFKSIE

250 260 270 280 290 300 310 320

| | | | | | | |

AcrBEc AQTRLTSTEEFGKILLKVNQDGSRVLLRDVAKIELGGENYDIIAEFNGQPASGLGIKLATGANALDTAAAIRAELAKMEP

MexBPa GKTRLQTAEQFENILLKVNPDGSQVRLKDVADVGLGGQDYSINAQFNGSPASGIAIKLATGANALDTAKAIRQTIANLEP

BesB EIGNVVIAYKIPDISSGINLSPIEIKLKDIANIKTDFEDLSEYVEYNGLPSISLSVQKRSDSNSIAVSNVVMNEIEKLKL

330 340 350 360 370 380 390 400

| | | | | | | |

AcrBEc FFPSGLKIVYPYDTTPFVKISIHEVVKTLVEAIILVFLVMYLFLQNFRATLIPTIAVPVVLLGTFAVLAAFGFSINTLTM

MexBPa FMPQGMKVVYPYDTTPVVSASIHEVVKTLGEAILLVFLVMYLFLQNFRATLIPTIAVPVVLLGTFGVLAAFGFSINTLTM

BesB SMPKDMKLEIASDSTDFIKASISTVVNSAYFGAMLAIFVIFFFLRSFRATIIIGISIPIAIVLTFCLMYFVNISLNIMSL

410 420 430 440 450 460 470 480

| | | | | | | |

AcrBEc FGMVLAIGLLVDDAIVVVENVERVMAEEGLPPKEATRKSMGQIQGALVGIAMVLSAVFVPMAFFGGSTGA---IYRQFSI

MexBPa FGMVLAIGLLVDDAIVVVENVERVMAEEGLSPREAARKSMGQIQGALVGIAMVLSAVFLPMAFFGGSTGV---IYRQFSI

BesB AGLALGIGMVVDCSIVVIDNIYKYR-QKGAKLISSSILGAQEMMLPITSSTFTSICVFGPFLIFKSELGVYGDFFKDFTF

490 500 510 520 530 540 550 560

| | | | | | | |

AcrBEc TIVSAMALSVLVALILTPALCAT---MLKPIAKGDHGEGKKGFFGWFNRMFEKSTHHYTDSVGGILRSTGRYLVLYLIIV

MexBPa TIVSAMALSVIVALILTPALCAT---MLKPIEKGDHGEHKGGFFGWFNRMFLSTTHGYERGVASILKHRAPYLLIYVVIV

BesB TIVISLGVSLLVAIFLVPVLSSHYVGLYTSFQKNIKNAFIRKIDAFFASIYYFLEFLYINLLNIVLNHKLIFGLIVFFSF

570 580 590 600 610 620 630 640

| | | | | | | |

AcrBEc VGMAYLFVRLPSSFLPDEDQGVFMTMVQLPAGATQERTQKVLNEVTHYYLTKEKNNVESVFAVNGFGFAGRGQNTGIAFV

MexBPa AGMIWMFTRIPTAFLPDEDQGVLFAQVQTPPGSSAERTQVVVDSMREYLLEKESSSVSSVFTVTGFNFAGRGQSSGMAFI

BesB IGSLLLGLLLDVTTFTRGKENSITINLNFPHKTNLEYAKFYSNRFLE-IVKSEAKGYKSIIATL--------RADRITFN

650 660 670 680 690 700 710 720

| | | | | | | |

AcrBEc SLKDWADRPGEENKVEAITMRATRAFSQIKDAMVFAFNLPAIVELGTATGFDFELIDQAGLGHEKLTQARNQLLAEAAKH

MexBPa MLKPWEERPGGENSVFELAKRAQMHFFSFKDAMVFAFAPPSVLELGNATGFDLFLQDQAGVGHEVLLQARNKFLMLAAQN

BesB VLFPLKEESRDNLTQSVDYDSIKYKIMNRIGNLYPEFNIEPSISGNALGGGDSIKIKISANDFEYIKDYGKILVSMLKKE

730 740 750 760 770 780 790 800

| | | | | | | |

AcrBEc PDMLTSVRPNGLEDTPQFKIDIDQEKAQALGVSINDINTTLGAAWGGSYVNDFIDRGRVKKVYVMSEAKYRMLPDDIGDW

MexBPa P-ALQRVRPNGMSDEPQYKLEIDDEKASALGVSLADINSTVSIAWGSSYVNDFIDRGRVKRVYLQGRPDARMNPDDLSKW

BesB IPELVNPRLSISDFQLQIGVEIDRALVYNYGIDMNTILNELKANINGVVAGQYVEKGLNYDIVLKLDRMDVKNLKDLEKI

810 820 830 840 850 860 870 880

| | | | | | | |

AcrBEc YVRAADGQMVPFSAFSSSRWEYGSPRLERYNGLPSMEILGQAAPGKSTGEAME-LMEQLASKLP--TGVGYDWTGMSYQE

MexBPa YVRNDKGEMVPFNAFATGKWEYGSPKLERYNGVPAMEILGEPAPGLSSGDAMA-AVEEIVKQLP--KGVGYSWTGLSYEE

BesB FITNSSGVKIPFSSIATFEKTNKAESIYRENQALTIYLNAGISPDDNLTQVTAKVVDFINNKVPHKEGITLKVEGEYNEF

890 900 910 920 930 940 950 960

| | | | | | | |

AcrBEc RLSGNQAPSLYAISLIVVFLCLAALYESWSIPFSVMLVVPLGVIGALLAATFRGLTNDVYFQVGLLTTIGLSAKNAILIV

MexBPa RLSGSQAPALYALSLLVVFLCLAALYESWSIPFSVMLVVPLGVIGALLATSMRGLSNDVFFQVGLLTTIGLSAKNAILIV

BesB SNIMNQFKIIIMMAIIVVFGIMASQFESFLKPFIIIFTIPLTAIGVVLIHFLAGEKLSIFAAIGMLMLVGVVVNTGIVLV

970 980 990 1000 1010 1020 1030 1040

| | | | | | | |

AcrBEc EFAKDLMDKEGKGLIEATLDAVRMRLRPILMTSLAFILGVMPLVISTGAGSGAQNAVGTGVMGGMVTATVLAIFFVPVFF

MexBPa EFAKELHE-QGKGIVEAAIEACRMRLRPIVMTSLAFILGVVPLAISTGAGSGSQHAIGTGVIGGMVTATVLAIFWVPLFY

BesB DYT-GLLIKRGFGLREAIIESCRSRLRPILMSSLTSIIGLIPMAFSSGSGNELLKPIAFTFIGGMTASTFLTLFFIPMLF

1050 1060

| |

AcrBEc VVVRRRFSRKNEDIEHNHTVDHH-

MexBPa VAVSTLF--KDEASKQQASVEKGQ

BesB EIFPTCFKFQI-------------
